# Template synthesis, DNA binding, antimicrobial activity, Hirshfeld surface analysis, and 1D helical supramolecular structure of a novel binuclear copper(ii) Schiff base complex†

**DOI:** 10.1039/d2ra00719c

**Published:** 2022-05-05

**Authors:** Hamid Goudarziafshar, Somaieh Yousefi, Yunes Abbasi Tyula, Michal Dušek, Václav Eigner

**Affiliations:** Department of Chemical Engineering, Hamedan University of Technology Hamedan Iran hamid_gafshar@yahoo.com; Department of Chemistry, Faculty of Science, Ilam University P.O. Box 69315516 Ilam Iran Abbasi_yunes@yahoo.com; Institute of Physics of the Czech Academy of Sciences Na Slovance 2, 182 21 Praha 8 Czech Republic

## Abstract

A new binuclear copper(ii) Schiff base complex [Cu_2_ L_2_^−^ (NO_3_)_2_]·2CH_3_OH (1) [*L* = 2,6-bis((*E*)-(*p*-tolylimino)methyl)-4-methoxyphenol] was synthesized using a template method in which the tridentate N_2_O Schiff base ligand was derived from [1 + 2] condensation of 2,6-diformyl-4-methoxyphenol and *p*-methyl aniline in the presence of copper(ii) ions as the template agent. The X-ray diffraction analyses revealed that this complex crystallizes in the monoclinic system with space group P2_1_/*n*. The most remarkable structural feature of 1 is that it contains two types of 1D right-handed helical chains. The molecules are linked by intermolecular hydrogen bonds and π⋯π interactions, then a 3D supramolecular network was constructed. Moreover, the intermolecular interactions on the crystal packing of 1 have been further studied using Hirshfeld surface analysis and corresponding 2D fingerprint plots. Binding interaction of this complex with calf thymus DNA (CT-DNA) has been investigated using absorption and emission studies, viscosity experiments and circular dichroism studies. Complex 1 shows significant binding to the DNA. The results of fluorescence spectroscopy and UV absorption spectroscopy, CD spectroscopy and viscosity indicated that this complex interacted with CT-DNA in a groove binding mode where the binding constant was 1.3 ± 0.2 × 10^4^ L mol^−1^. Our fluorimeteric study showed that the reaction between 1 and CT-DNA was exothermic (Δ*H* = 59.6 kJ mol^−1^; Δ*S* = 268.79 J mol^−1^ K^−1^). Antibacterial activities of the complex were screened by the disc diffusion method against three Gram-positive bacteria (*Staphylococcus aureus* ATCC 25923, *Enterococcus faecalis* ATCC 23212 and *S. epidermidis* ATCC 34384), and three Gram-negative bacteria (*Escherichia coli* ATCC 25922, *Pseudomonas aeruginosa* ATCC 27853 and *Klebsiella pneumonia* ATCC 70063). The results indicated that this complex demonstrated acceptable antibacterial activities.

## Abstract 2

A new binuclear copper(ii) Schiff base complex was synthesized using a template method in which the tridentate N_2_O Schiff base ligand was derived from [1 + 2] condensation of 2,6-diformyl-4-methoxyphenol and *p*-methyl aniline.

## Graphical abstract

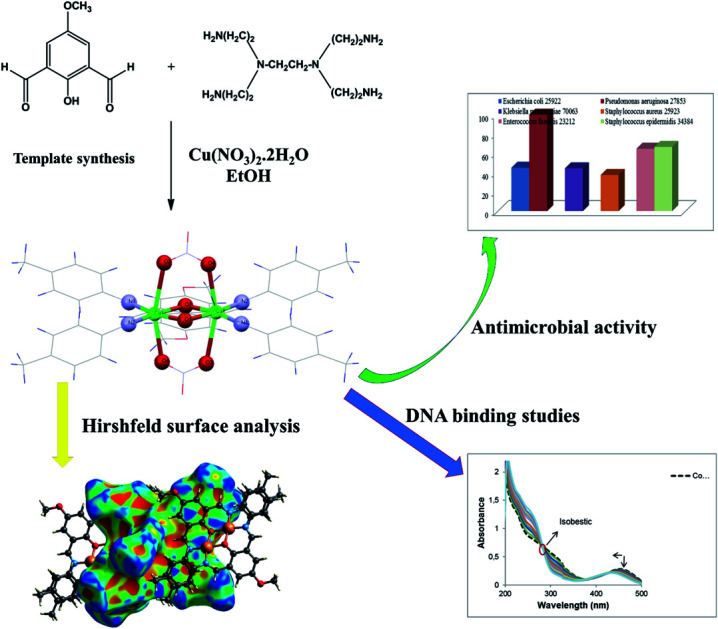

## 1. Introduction

Helical structures retain a unique place in life since genetic information is encoded within helical DNA molecules. Much effort has been devoted in recent years to the design and fabrication of metal complexes exhibiting helical architectures, not only in modeling of biological systems, but also for applying them in the fields of supramolecular chemistry, asymmetric catalysis, and nonlinear optical material,^1–8^ and the aesthetically appealing structural topology. Reviews have shown the significance of such complexes^9^ and structures with a single helix,^10^ double helices,^11^ and triple helices,^12^ whether ligand directed,^13^ spacer controlled,^14^ or anion induced,^15^ employing many different kinds of N, O, and/or S containing coordinating ligands. Such helical molecules have been designed and assembled by the selection of basic components such as the coordination geometry of metal ions, the binding site of donating atoms, and the length of spacers.^16–18^ Single stranded helical structures are the most commonly observed form compared to double and triple stranded structures, either with enantiopure or achiral ligands. The secondary structure of helical complexes is determined by weak metal–ligand covalent interactions, halide ions such as iodide and supramolecular interactions such as hydrogen bonding and π⋯π stacking.^19–24^ The design and synthesis of small synthetic systems that can recognize specific sites of DNA through formation of non-covalently associated complexes are important areas of much current interest.^25^ Such physical complexation, more often, may produce important pharmacological effects by interfering with the biological processes in which DNA/RNA takes part. Such investigations have also provided insights as to the mechanism of action for antitumor antibiotics.^26^ The metal complexes can bind to DNA by different connection modes, such as π⋯π interactions which happen when the ligand contains planar heterocyclic ring systems, or when there is groove binding in large molecules through hydrogen bonding and van der Waals interactions.^27^ Beside, metal complexes of Schiff-bases have been emerged for the development of efficient variety of science.^28–32^

In the present study, we reported crystal structure of helical binuclear copper(ii) Schiff base complex, [Cu_2_L_2_^−^(NO_3_)_2_]·2CH_3_OH (1) [*L* = 2,6-bis((*E*)-(*p*-tolylimino)methyl)-4-methoxyphenol] containing two types of 1D right-handed helical chains. We also examined the close intermolecular interactions between the molecules in the solid state of the crystal using Hirshfeld surface analysis. In addition, antibacterial activity of complex was investigated. Furthermore, DNA binding of 1 was studied using different physico-chemical methods. A list of abbreviations used in the text with explanations shows in Table S1.†

## 2. Experimental

### 2.1. Materials

High purity *p*-methyl aniline, copper(ii) nitrate hydrate, 4-methoxy phenol, hexamethylenetetramine, trifluoroacetic acid were purchased from commercial sources and used as received. 2,6-Diformyl-4-methoxy phenol was synthesized by the reported procedure.^51^ Commercially pure chemicals such as Tris–HCl (Sigma Company, Madrid, Spain), were provided and used without purification. Highly polymerized calf thymus-DNA (CT-DNA) was procured from Sigma Company. All the experiments of DNA interaction were carried out in Tris–HCl buffer solutions and double distilled water (10 mM, pH 7.4). The stock solution of CT-DNA was procured by dissolving approximately 1–2 mg of CT-DNA fibers in 2 mL Tris–HCl buffer and stored at 4 °C for 24 hours. The concentration of DNA in stock solution was expressed in monomer units, as determined by spectrophotometry at 260 nm using the extinction coefficient (*ε*_p_) of 6600 M^−1^ cm^−1^. DNA solutions were employed after no more than 4 days.

### 2.2. Instrumentation

In order to characterize and assess the structure of 1, the following instruments were used: the C, H, N data were obtained by using a Costech ECS 4010 elemental analyzer. FT-IR spectra were recorded on Bruker Vertex 70 spectrometer in KBr pellets from 400–4000 cm^−1^. The absorbance spectra were recorded for 1 using an HP spectrophotometer (Agilent 8453) equipped with a thermostated bath (Huber polysat cc1). The absorbance measurements were performed by keeping the complex concentration constant (2.5 × 10^−5^ M) while varying the DNA concentration from 0 to 8.0 × 10^−5^ M (*r*_i_ = [DNA]/[complex] = 0.0–3.2). The spectra were recorded in the range of 200–500 nm. CD measurements were recorded on a JASCO (J-810) spectropolarimeter, keeping the concentration of DNA constant (5 × 10^−5^ M) while varying the complex concentration from 0 to 3.0 × 10^−5^ M (*r*_i_ = [complex]/[DNA] = 0.0–0.6). Viscosity measurements were made using a viscosimeter (SCHOT AVS 450) preserved at 25 °C 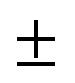 0.5 °C in a constant temperature bath while the DNA concentration was fixed at 5 × 10^−5^ M and flow time was admeasured with a digital stopwatch. The data is reported as (*η*/*η*_0_)^1/3^*versus r*_i_ (*r*_i_ = [complex]/[DNA] = 0.0–1.0), where *η*_0_ is the viscosity of the DNA solution alone. All fluorescence measurements were performed on a JASCO spectrofluorimeter (FP6200) using a quartz cell of 1 cm path length by keeping the concentration of the complex constant (1 × 10^−5^ mol L^−1^) while varying the DNA concentration from 0 to 3.4 × 10^−5^ M at three different temperatures (293, 310, 318 K). The samples were excited at 260 nm and the emission spectra were recorded in the range of 300 to 480 nm. The quenching experiments were conducted by adding stoichiometric small aliquots of potassium iodide stock solution (0.1 mol L^−1^) to 1 and CT–DNA–Cu complex solutions, respectively. The fluorescence intensity was recorded and the quenching constants (*k*_sv_) were calculated.

### 2.3. Crystallographic measurements

Single crystal X-ray diffraction intensity data of 1 was performed at 120 K using Gemini four-circle diffractometer from Agilent Technologies, equipped with a mirror collimated Cu Kα radiation (1.54184 Å) and area detector Atlas. The structure was solved by direct methods using SUPERFLIP program^33^ and refined by full-matrix least-square technique on *F*^2^, using JANA 2006 (ref. 34) program with anisotropic displacement parameters for all non-hydrogen atoms. All hydrogen atoms were discernible in difference Fourier maps and could be refined to reasonable geometry. According to common practice, H atoms bonded to C were kept in ideal positions while positions of H atom bonded to O were refined with restrained geometry. In both cases *U*_iso_(H) was set to 1.2*U*_eq_(C,O). The molecular structure plots were prepared using the ORTEP III^35^ and Mercury.^36^ Crystallographic data and refinement parameters are summarized in Table 1, and the selected bond lengths and angles for 1 are displayed in Table 2. The selected hydrogen bonds are presented in Table 3.

**Table 1:** Crystal data and structure refinement of 1

Formula	C_46_H_42_Cu_2_N_6_O_10_ · 2(CH_4_O)
CCDC no	1456947
Formula weight	1030
Temperature (K)	120
Wavelength (Å)	1.5418
Crystal system, space group	Monoclinic, *P*2_1_/*n*
Unit cell dimensions	
*a*, *b*, *c* (Å)	8.3185 (4), 17.6483 (7), 15.7257 (8)
β/(°)	101.951 (4)
Volume (Å^3^)	2258.61 (18)
*Z*, *D*_calc_/(Mg m^−3^)	2, 1.515
Absorption coefficient (mm^−1^)	1.78
*F*(000)	1068
Crystal size (mm^3^)	0.30 × 0.25 × 0.14
Crystal shape/colour	Block, black
*θ* range for data collection/(^°^)	5.0–66.7
Limiting index	−9,9/-14,20/-13,18
Reflections collected/unique	3911/3512 [*R*(int) = 0.023]
Absorption correction	Analytical
Data/restraints/parameters	3911/3/310
Goodness-of-fit on *F*^2^	97
Final *R* indices [*I* > 3*σ* (*I*)]	*R*1 = 0.041, w*R*_2_ = 0.111
*R* Indices (all data)	*R*1 = 0.045, w*R*_2_ = 0.114
Largest diff. peak, hole (e Å^−3^)	0.58 and −0.78

**Table 2:** Selected bond lengths (Å) and angles (°) for 1^a^

Bond length	
Cu1–O1	1.9529 (17)
Cu1–O1^i^	1.9647 (16)
Cu1–N1^i^	1.970 (2)
Cu1–N2	1.975 (2)
Cu1–O3^i^	2.655 (18)
Cu1–O4	2.504 (16)

Bond angle	
O1–Cu1–O1^i^	78.82 (7)
O1–Cu1–N1^i^	167.94 (8)
O1–Cu1–N2	90.55 (8)
O1^i^–Cu1–N1^i^	90.30 (8)
O1^i^–Cu1–N2	169.36 (8)
N1^i^–Cu1–N2	100.32 (8)
O4–Cu1–O3^i^	160.85 (57)
O3^i^–Cu1–N1^i^	91.34 (67)
O4–Cu1–O1^i^	84.13 (62)
O4–Cu1–O1	85.41 (62)
O4–Cu1–N1^i^	98.75 (67)
O4–Cu1–N2	94.76 (68)
O3^i^–Cu1–O1	81.67 (6)
O3^i^–Cu1–O1^i^	79.56 (61)
O3^i^–Cu1–N2	99.41 (7)

a Symmetry code: (i) −*x*, −*y* + 2, −*z*.

**Table 3:** Intra and intermolecular hydrogen bonds in 1^a^

D–H⋯A	D–H	H⋯A	D⋯A	D–H⋯A
C13–H1*c*13⋯O3	0.96	2.349 (17)	3.240 (3)	154.06 (15)
C17–H1*c*17⋯O4	0.96	2.31 (17)	3.203 (3)	153.82 (16)
O1*m*–H1*o*1*m*⋯O5	0.834 (19)	1.963 (18)	2.749 (3)	156.8 (17)
C4–H1*c*4⋯O1m	0.96	2.698 (29)	3.549 (37)	148.04 (16)
C15–H1*c*15⋯O1m	0.96	2.519 (26)	3.349 (35)	144.59 (15)
C21–H1*c*21⋯O1m	0.96	2.675 (30)	3.592 (39)	159.78 (15)
C7–H1*c*7⋯O5^ii^	0.96	2.38 (2)	3.284 (3)	157.00 (15)
C6–H1*c*6··· O5^ii^	0.96	2.53 (17)	3.384 (3)	148.17 (15)
C22–H2*c*22⋯O2	0.96	2.59 (17)	3.499 (33)	157.61 (18)
C20–H1*c*20⋯N3	0.96	2.596 (2)	3.384 (32)	139.52 (15)

a Symmetry code: (ii) −*x* − 1, −*y* + 2, −*z*.

### 2.4. Hirshfeld surfaces analysis

Hirshfeld Surface (HS) analysis has become an attractive and growing technique in description of crystal structures.^37–40^ HS and the associated 2D fingerprint plots (FP) have been generated using Crystal Explorer 3.0 program.^41^ When the structure input file in the CIF format was uploaded into the CrystalExplorer software, all bond lengths to hydrogen were automatically modified to typical standard neutron values^42^ (C–H = 1.083 A, N–H = 1.009 A and O–H = 0.983 A). The 2D fingerprint plots were represented by using the standard 0.6–2.4 Å view with the *d*_e_ and *d*_i_ distance scales displayed on the graph axes, where *d*_e_ is the distance from the Hirshfeld surface to the nearest nucleus outside the surface and *d*_i_ is the corresponding distance to the nearest nucleus inside the surface. The function *d*_norm_ is a ratio that comprises the *d*_e_, *d*_i_ and the van der Waals radii (*r*_vdW_) of the atom. The *d*_norm_ values were mapped onto the Hirshfeld surface using a red-blue-white colour scheme as follows: red regions with the negative values of *d*_norm_ represent the intermolecular contacts shorter than the sum of the van der Waals radii; blue regions with positive values of *d*_norm_ represent the intermolecular contacts longer than the sum of the van der Waals radii; white regions denote the distance of contacts exactly corresponding to the van der Waals separation with a *d*_norm_ values of zero.^43^ Furthermore, two colored properties namely, shape-index and curvedness surfaces can be specified based on the local curvature of the Hirshfeld surface, which indicates the interactions between neighboring molecules.^44^

### 2.5. Antimicrobial activity

The cultures of *Staphylococcus epidermidis* ATCC 34384*, Staphylococcus aureus* ATCC 25923 *Enterococcus faecalis* ATCC 29212, *Escherichia coli* ATCC 25922, *Pseudomonas aeruginosa* ATCC 27853, and *Klebsiella pneumonia* ATCC 70063 were obtained from Hacettepe University, Department of Medical Microbiology. Bacterial strains were cultured overnight at 37 °C in nutrient broth. During the survey, these stock cultures were stored in the dark at 4 °C.

#### 2.5.1 Disc diffusion method

For the investigation of the antibacterial activity, the synthesized complex, 1, was dissolved in dimethylsulfoxide (20% DMSO) to a final concentration of 10 mg mL^−1^ and sterilized by filtration through 0.45 μm millipore filters. Antimicrobial tests were then carried out by the disc diffusion method using 100 μL of suspension containing 108 CFU mL^−1^ bacteria, which was spread on a nutrient agar (NA) medium. The discs (6 mm in diameter) were impregnated with 25 μL of compound (200 μg per disc) at the concentration of 8 mg mL^−1^ and placed on the inoculated agar. DMSO impregnated discs were used as negative control. Sulfioxazole (300 μg per disk) were used as positive reference standards to determine the sensitivity of one strain/isolate in each microbial species tested. The inoculated plates were incubated at 37 °C for 24 h for bacterial strains isolates. The antimicrobial activity in the disc diffusion assay was examined by measuring the zone of inhibition against the test organisms. Each assay in this experiment was repeated twice.^45,46^

#### 2.5.2 Micro dilution assays

The minimal inhibition concentration (MIC) values, except one, were also studied for the microorganisms sensitive to compound determined in the disc diffusion assay. The inocula of microorganisms were prepared from 12 h broth cultures and suspensions were adjusted to 0.5 McFarland standard turbidity. The test compound dissolved in dimethylsulfoxide (DMSO) was first diluted to the highest concentration (2000 μg mL^−1^) to be tested, and then serial, two-fold dilutions were made in a concentration range from 15.625 to 2000 μg mL^−1^ in 10 mL sterile test tubes containing nutrient broth. The MIC values of compound against bacterial strains were determined based on a micro-well dilution method.^47^ The 96-well plates were prepared by dispensing 95 μL of nutrient broth and 5 μL of the inoculums into each well. 100 μL from the test compound initially prepared at the concentration of 2000 μg mL^−1^ was added into the first wells. Then, 100 μL from each of their serial dilutions was transferred into eight consecutive wells. The last well containing 195 μL of nutrient broth without compound, and 5 μL of the inoculums on each strip, was used as negative control. The final volume in each well was 200 μL. The contents of the wells were mixed and the micro plates were incubated at 37 °C for 24 h. The compound tested in this study was screened twice against each microorganism. The MIC was defined as the lowest concentration of the compounds to inhibit the growth of microorganisms.^48^

### 2.6. Synthesis of [Cu_2_L_2_^−^(NO_3_)_2_]·2CH_3_OH (1)

A methanolic solution (10 mL) of *p*-methyl aniline (0.107 g, 1 mmol) was added dropwise to a solution of Cu(NO_3_)_2_·2H_2_O (0.162 g, 0.5 mmol) and 2,6-diformyl-4-methoxy phenol (0.09 g, 0.5 mmol) in the same solvent (15 mL). The mixture of reaction was heated for 24 h. Then, the deep green solution was kept in air for slow evaporation. After about a week, dark green colored crystals appeared. Yield: 0.722 g (70%), anal. calc. for C_46_H_42_Cu_2_N_6_O_10_, 2(CH_4_O): C, 55.97; H, 4.89; N, 8.16. Found: C, 56.00; H, 4.90; N, 8.16%. *Λ*_M_: (1 × 10^−3^ M in DMF) 73.86 Ω^−1^ cm^2^ mol^−1^). IR (KBr, cm^−1^): 3408 (O–H), 2831 (C–H), 1624, 1614 (C=N), 1584 (C=C); UV-vis [*λ*_max_, 454 nm, 270 nm (lit mol^−1^cm^−1^). MS (EI): (*m*/*z*) = 840 [Cu_2_L_2_]^+^, 814 [Cu_2_L_2_–OMe + 2H^+^]^+^, 747 [L_2_ + CH_3_OH]^+^

## 3. Results and discussion

### 3.1. Synthesis and characterization of [Cu_2_ L_2_^−^ (NO_3_)_2_]. 2CH_3_OH (1)

1 was synthesized using reaction among Cu(ii) salt, 2,6-diformyl-4-methoxyphenol and *p*-methyl aniline in methanol solvent at reflux conditions. The exact coordination sphere of 1 was determined by single-crystal X-ray crystallography. In FT-IR spectra, the peaks at 1614 and 1624 cm^−1^ is due to C=N stretch.^49^ Conductivity measurements performed in DMF (Molar Conductance, *Λ*_M_ (1 × 10^−3^ M in DMF) 73.86 Ω^−1^ cm^2^ mol^−1^) confirm the non-electrolyte nature of the complex.^50^1 was stable in air at room temperature and soluble in common polar organic solvents such as ethanol, methanol, DMF, and DMSO. Moreover, the 1 was found to be stable in the solution phases used, as evidenced by the FT-IR spectra (Fig. S1†).

### 3.2. Electronic absorption spectroscopy

The electronic spectrum of 1 was recorded in EtOH. The maximal absorption peaks at 270 nm and 454 nm arising from intraligand π–π* transitions and charge transfer transition, respectively.^51–53^ Also, the weak band around 658 nm for 1 is assignable to the ^2^E_g_–^2^T_2g_ transition that is characteristic of octahedral geometry around Cu(ii) ion.^54^

### 3.3. Description of crystal structure [Cu_2_ L_2_^−^ (NO_3_)_2_]·2CH_3_OH (1)

**Fig. 1:**
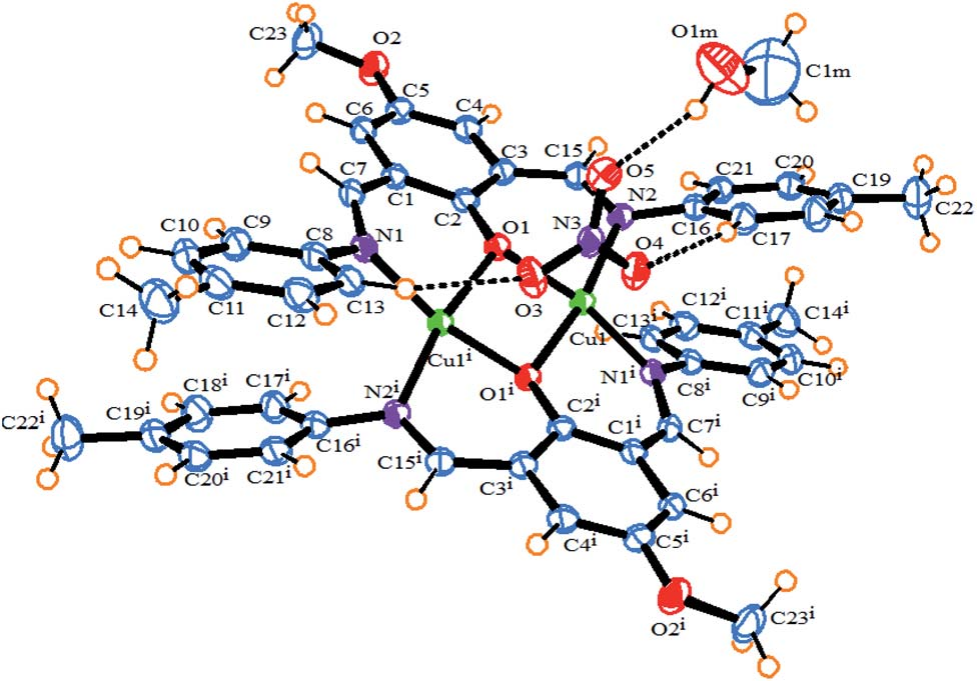
An ORTEP diagram of 1 with thermal ellipsoids at 55% probability. Inter and intra-molecular hydrogen bonds shown as dotted lines.

**Fig. 2:**
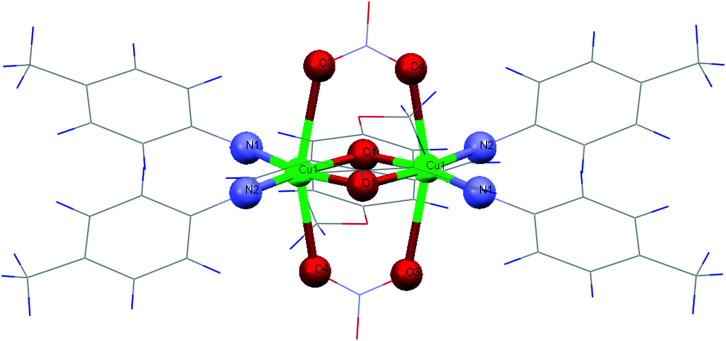
Coordination environments of the Cu(ii) in 1.

The ORTEP diagram of 1 is shown in Fig. 1. The crystallographic parameters, selected bond distances and angles around the metal centers, and selected hydrogen bonds are listed in Tables 1–3, respectively. The X-ray determination result of 1 reveals that it crystallizes in a monoclinic crystal system with space group P2_1_/*n* and each asymmetric unit contains half of the crystallographically unique molecule (Fig. 1). Each Cu(ii) center displays a six-coordinate distorted octahedral geometry with CuN_2_O_4_ configuration (Fig. 2), where the basal plane is composed of two phenoxo-bridged μ_2_-O1 atoms and two imino nitrogen atoms (N1 and N2) from two deprotonated Schiff base ligands (L^−^). Moreover, the axial positions are occupied by the two bridging oxygen atoms from two nitrate anions (O3 and O4). In the CuN_2_O_4_ coordination sphere, the axial Cu–O contacts of μ_2_-NO_3_ bridged anions (Cu–O3, 2.655 (18) Å) and Cu–O4, 2.504 (16) Å) are much longer than (Cu–O1, 1.965 (2) Å and Cu–O1, 1.953 (2) Å), demonstrating the Jahn–Teller effect^55^ (Table 2). The Cu–N bond lengths in this complex are 1.970 and 1.975 and are similar to a previously reported six-coordinated copper(ii) complex with nitrogen donor atoms.^56–59^

Also, the bond lengths and angles of 1 are in close agreement with those reported for other six-coordinated copper(ii) complexes.^60,61^ The distance of dinuclear center is 3.027(5) Å. This long distance between two Cu(ii) centers suggests that there is no interaction between these two metal centers.^61^ We found two intermolecular C–H⋯O hydrogen bonds between the hydrogen atoms of aromatic rings of ligands and oxygen atoms of bridging nitrate anions as well as one intramolecular π⋯π stacking interaction between the aromatic rings of two ligands with a distance of 3.423 Å in the complex which, play an important role in stabilizing the structure. The O⋯H distances and C–H⋯O angles are 2.349 (17) Å, 154.06° for C13–H1⋯O3 and 2.31 (17) Å, 153.82° for C17–H1⋯O4, respectively. As shown in Fig. S2b,† the non-coordinated oxygen atom O(5) of the bridging NO_3_^−^ forms bifurcated hydrogen bonds with the hydrogen atoms attached to the imino and phenyl carbon atoms of Schiff base ligand, C7–H1⋯O5 and C6–H1⋯O5, respectively (Table 3). Furthermore, there are π–π stacking interactions between the aromatic rings of Schiff base ligands belonging to neighboring units in complex 1, leading to the neighboring complex molecules connected together along the *a* axis (Fig. S2a†).

**Fig. 3:**
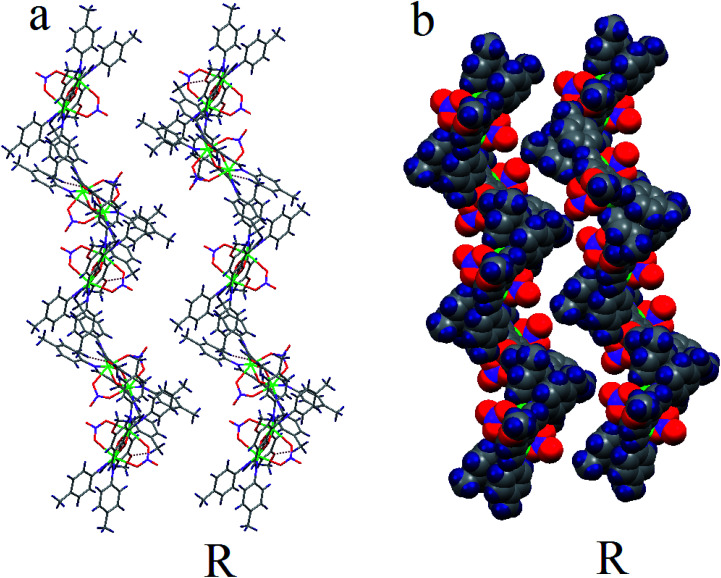
(a) Hydrogen bonds network. (b) Space-filling view of the 1D right-handed (R) helical chains along *b* axis.

This 1D chain structures which are formed along the *a* axis, are linked together through C–H⋯N (C20–H1⋯N3 2.596(2) Å, C–H⋯O (C4–H1⋯O1m 2.698(29) Å, C15–H1⋯O1m 2.519(26) Å, C21–H1⋯O1m 2.675(30) Å) and O–H⋯O hydrogen bonds (O1*m*–H1⋯O5 2.614(4) Å) to form 2D supramolecular structure (Fig. S2b†). The resulting 2D structures are joined into the 3D packing framework connected by C–H⋯O (C22–H2⋯O2 2.59(17) Å) (Fig. S2c†). The most remarkable structural feature of 1 is that it contains two types of 1D right-handed helical chains. One is along the *b* axis, whose the [Cu_2_ L_2_^−^ (NO_3_)_2_] monomers are connected through C–H⋯O hydrogen bonds between the C–H group of a phenyl ring and O-methoxy group of Schiff base ligands (C22–H2⋯O2 2.59(17) Å) belonging to neighboring units in complex 1 (Fig. 3). The other is along the perpendicular to *ac* plane (b* axis) by the hydrogen bonding between the nitrogen atom of the coordinated nitrate group and C–H group of a phenyl ring Schiff base ligand (C20–H1⋯N3 2.596(2) Å) (Fig. S3†).

### 3.4. Hirshfeld surface analysis of [Cu_2_ L_2_^−^ (NO_3_)_2_]·2CH_3_OH (1)

**Fig. 4:**
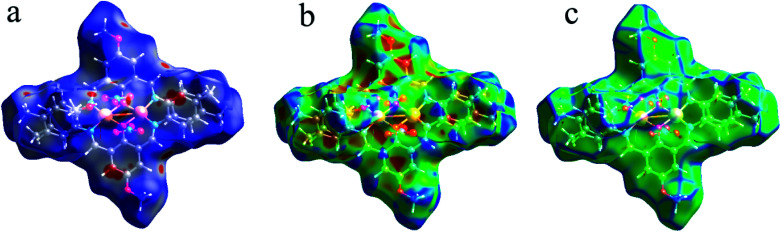
Hirshfeld surfaces mapped with (a) *d*_norm_, (b) shape index and (c) curvedness for 1.

**Fig. 5:**
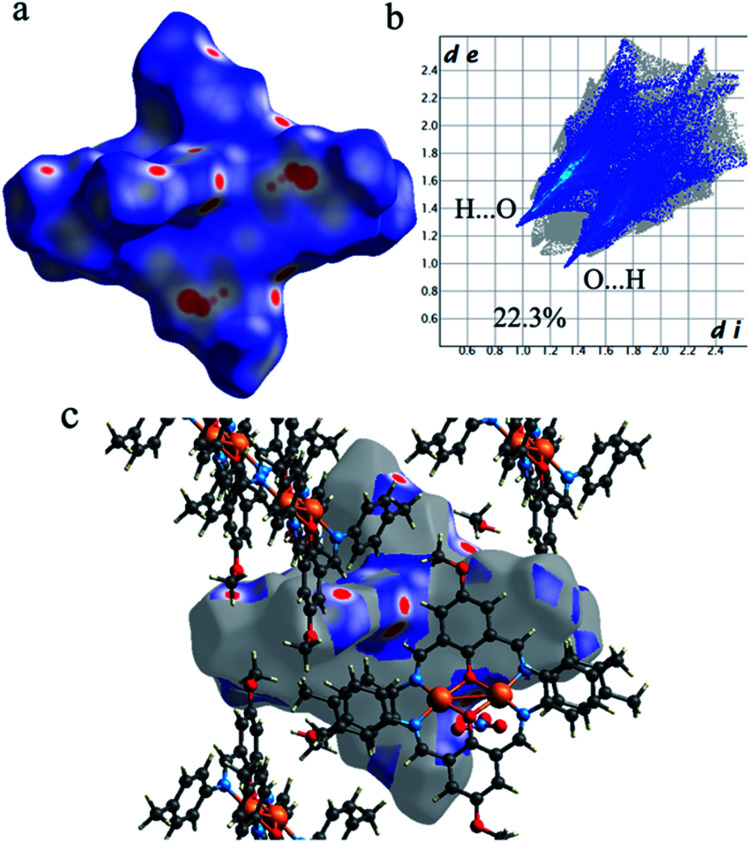
(a) The Hirshfeld surfaces mapped with *d*_norm_ function, (b) the Hirshfeld surfaces mapped with *d*_norm_ function and (c) corresponding 2D fingerprint for the C–H⋯O interactions in 1.

As shown in Fig. 4, the Hirshfeld surfaces (*d*_norm_, shape index, and curvedness) of 1 are mapped over *d*_norm_ (ranging from −0.303 (red) to 1.370 (blue) Å), shape index (ranging from −1 to 1.0 Å), and curvedness (ranging from −4.0 to 0.4) Å. The molecular surfaces of 1 are shown as transparent to allow the visualization of the molecular moiety, around which they were calculated. The red spots on the *d*_norm_ surface of 1 indicate the intermolecular close contacts involved in the hydrogen bonds such as, C–H⋯O (Fig. 5), N–H⋯O, while the white regions represent contacts around the van der Waals separation looking like H⋯H interactions and the blue areas are devoid of such close contacts. The intermolecular interactions involved in the molecular structure are visible on the 2D fingerprint plots, which can be decomposed to quantify particular contributions of intermolecular interactions in the molecular packing of a solid network.

**Fig. 6:**
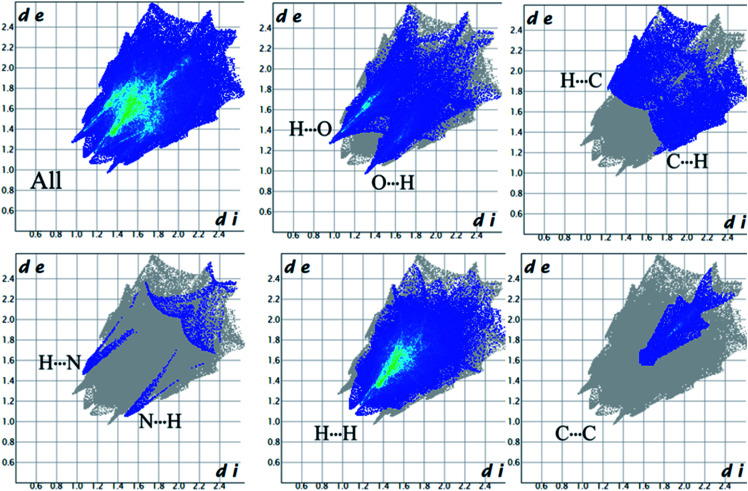
The 2D fingerprint plots for all interactions present in 1.

Complementary regions are evident in the fingerprint plots, indicating that a molecule acts as a donor (*d*_e_ > *d*_i_) and another one as an acceptor (*d*_e_ < *d*_i_). The 2D fingerprint plots of 1 show that the main intermolecular interactions between its molecules are H⋯H, H⋯N, H⋯O, H⋯C, and C⋯C intermolecular interactions (Fig. 6).

**Fig. 7:**
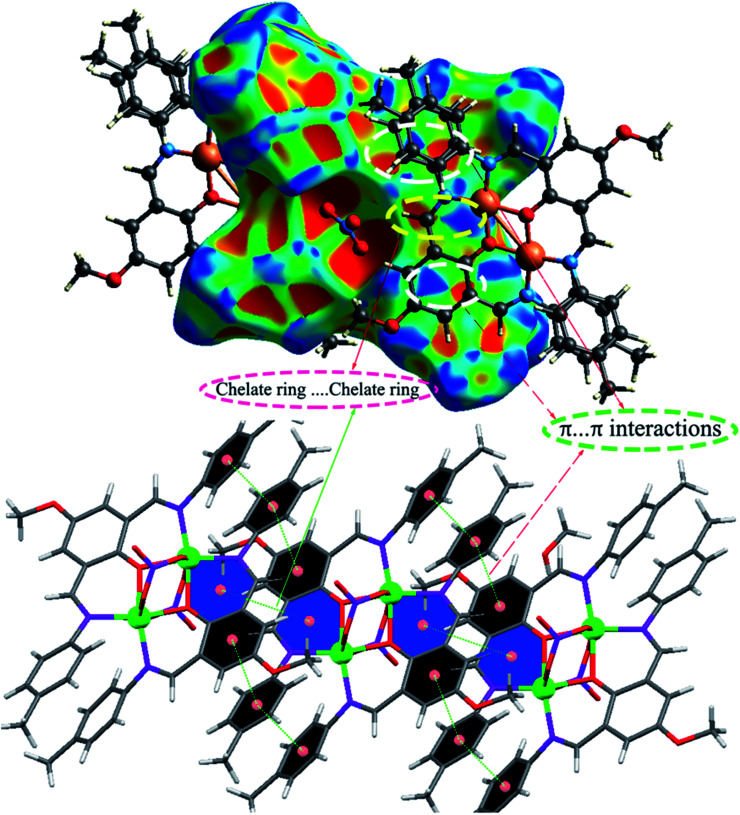
The Hirshfeld surfaces mapped with shape index function for the C⋯C (π⋯π) and chelate ring⋯chelate ring stacking interactions in 1.

Moreover, the O⋯H/H⋯O, and N⋯H/H⋯N interactions are represented by a spike in the bottom right (acceptor) area (O⋯H 8.5%, N⋯H 0.8%) and a spike at the top left (donor) area (H⋯O 13.8%, H⋯N 0.9%) of the 2D fingerprint plot. The H⋯C/C⋯H intermolecular interactions comprise 5.9% of the total Hirshfeld surface area which emerge as very short spike at the top left (H⋯C 6.2%) and bottom right (C⋯H 7.6%) of the 2D fingerprint plot. The H⋯H contacts appear in the largest region accompanied by the short and tenuous spike at the middle of fingerprint plot, which has the most significant contribution to the total Hirshfeld surfaces (53.7%). The 2D fingerprint plots of 1 show that the π⋯π (C⋯C) stacking interactions comprise 5.8% of the total Hirshfeld surface area. Furthermore, the presence of π⋯π (C⋯C) stacking interactions in the crystal packing of 1 are clearly confirmed by characteristic ‘bow-tie’ patterns in the shape index function (Fig. 7). Indeed, in the shape index, the highlighted red and blue triangles (highlighted circles in Fig. 7) prove the presence of the π⋯π stacking interactions in the crystal structure of 1, where the red triangle denotes the aromatic ring atoms of the molecule outside the surface, and the blue one represents the aromatic ring atoms inside the surface.

**Fig. 8:**
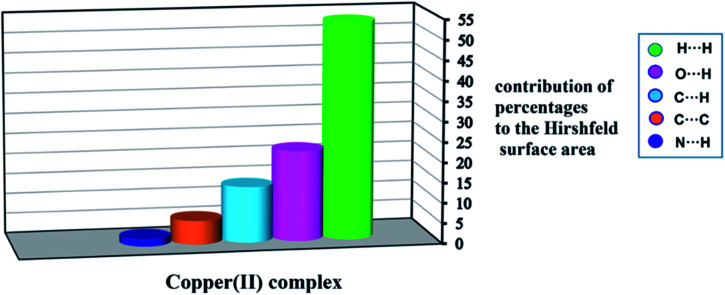
Relative contributions of various intermolecular interactions contributing to the Hirshfeld surfaces of 1.

The quantities of interactions with percentages are shown as a histogram in Fig. 8. Fig. 8 shows the H⋯H and N⋯H contacts provide the highest and lowest share of the total intermolecular interactions in the title complex.

### 3.5. Antibacterial activity results

The test compound was screened *in vitro* for their antibacterial activity against three Gram-negative species (*Escherichia coli*, *Pseudomonas aeruginosa*, *Klebsiella pneumonia*) and three Gram-positive species (*Staphylococcus epidermidis*, *Staphylococcus aureus*, *Enterococcus faecalis*) of bacterial strains by the disc diffusion and micro dilution methods. The antibacterial activities obtained by the two methods are given in Tables 4 and 5.

**Table 4:** Measured inhibition zone diameter (mm) of 1 and antibiotic by disc diffusion method^a^

Diameter inhibition zone (mm, 200 μg per disk)						
Bacteria strains	*E. coli* ATCC 25922	*P.aeruginosa* ATCC 27853	*K. pneumoniae* ATCC 70063	*S. aureus* ATCC 25923	*E. faecalis* ATCC 23212	*S. epidermidis* ATCC 34384
Cu complex	10	8	12	10	12	11
Sulfisoxazole	20	9	20	24	17	15

a Sulfisoxazole (300 μg per disk) < 10: weak; >10 moderate; >16: significant.

**Table 5:** The MICs of antibacterial activity of 1

Bacteria strains	MIC μg mL^−1^ (mM)					
	*E. coli* ATCC 25922	*P. aeruginosa* ATCC 27853	*K. pneumoniae* ATCC 70063	*S. aureus* ATCC 25923	*E. faecalis* ATCC 23212	*S. epidermidis* ATCC 34384
Cu complex	250 (0.446)	250 (0.446)	62.5 (0.111)	250 (0.446)	125 (0.223)	125 (0.223)
Sulfisoxazole	23.4 (0.088)	375 (1.403)	23.4 (0.088)	23.4 (0.088)	93.75 (0.35)	93.75 (0.35)

The disc diffusion assay results demonstrate (Table 4) that 1 has exhibited the moderate inhibition effect against most of the test bacteria (Fig. S4†). The ligands derived from the different amines and derivatives of salicylaldehyde have higher antibacterial activity than their respective complexes.^62^ The remarkable activity of such ligands may arise from existence of two hydroxyl groups in these ligands which may play an important role in antibacterial activity. However, Cu(ii) complex (1) in this paper has no hydroxyl group in its structure and has a lower antibacterial activity than similar Cu(ii) Schiff base complex in previous works.^63,64^

### 3.6. DNA binding studies

#### 3.6.1. Electronic absorption spectra

**Fig. 9:**
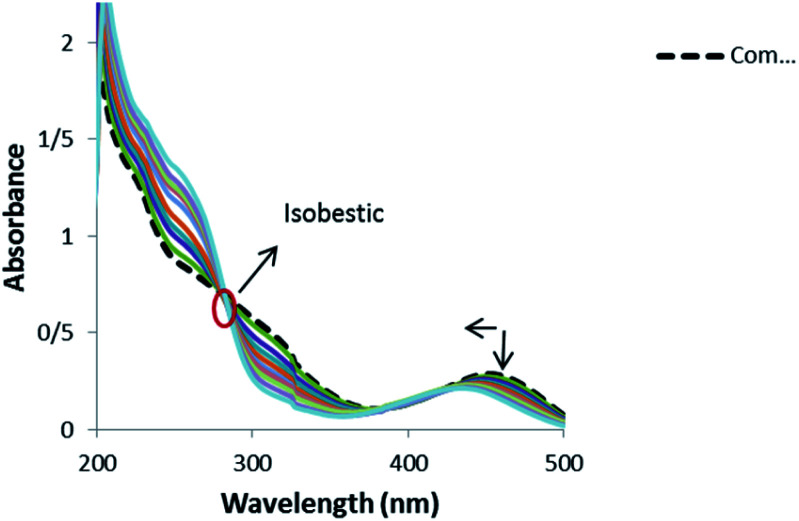
Absorption spectra of 1 (1.25 × 10^−5^ M) in the absence and presence of increasing amounts of CT-DNA: *r*_i_ = [CT-DNA]/[complex]: 0.0, 0.05, 0.1, 0.13, 0.17, 0.21, 0.25, 0.29, 0.33, 0.37, 0.65.

Electronic absorption spectroscopy is one of the most useful techniques for studying binding mode of metal complexes to DNA.^65,66^ Absorption spectra of 1 in the absence and presence of DNA (pH 7.4) showed a shoulder in the 200–350 nm regions and a peak at range of 400–500 nm (Fig. 9).

Band 454 gently decreased with the increasing concentration of DNA and a blue shift was observed from 454 to 433 nm. Also, an isobestic point at 280 nm provided evidence for the new complex–DNA formation.^67^ These observations offer that there exists a strong interaction between the complex and DNA. No red shift was observed in the UV spectra, which indicates that the binding mode is not intercalation and the hypochromism indicates that the binding mode of 1 to DNA might be groove binding. The similar hypochromism without red shift was observed for the complexes of copper(ii) and nickel(ii) containing the drug mesalamine that bound to DNA through groove mode.^68,69^

In order to further illustrate the binding strength of 1 with CT-DNA, the intrinsic binding constant *K*_b_ was determined from the spectral titration data using the following equation:1
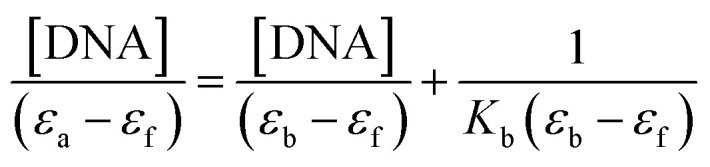
where, [DNA] is the concentration of DNA. *ε*_a_, *ε*_f_ and *ε*_b_ correspond to *A*_obs_/[compound], the extinction coefficient for the free complex and its entirely DNA-bound combination, respectively. In the plots of [DNA]/(*ε*_a_ _ *ε*_f_) *versus* [DNA], *K*_b_ was obtained by the ratio of the slope to intercept (Fig. S5†). The binding constant, *K*_b,_ for 1 was 1.3 ± 0.2 × 10^4^ L mol^−1^. This observation indicated the binding capability of the complex toward DNA with an affinity lower than the classical intercalators, due to the two chlorides with the DNA base pairs and relatively bulky structure of ligand molecules. Such a bulky structure cannot allow them to stack between the base pairs and inhibits any other bond formation (hydrogen bonds, *etc.*) with DNA.^70^ This *K*_b_ value is similar to those reported for well-established groove binding agent ([Ni(phen)_3_]^+2^, 1.4 × 10^4^ M^−1^),^71^ ([Ho(phen)_2_Cl_3_]·H_2_O (1.36 × 10^4^ M^−1^),^72^ (Cu–Sn_2_ complex, 1.67 × 10^4^ M^−1^).^73^ It was detected that the binding mode between the complex and DNA was groove binding.

#### 3.6.2. Viscosity measurements

Hydrodynamic methods that are sensitive to length change (*i.e.*, viscosity and sedimentation) are considered to be one of the least ambiguous and most exacting tests of a binding mode in solution in the absence of crystallographic structural data. The sensitivity of viscosity measurement largely depends on the changes in the length of DNA that occur as a consequence of its different binding modes with guest molecules. Intercalating agents of ligand such as the classical intercalations are expected to elongate the double helix to incorporate the small molecules in between the bases leading to an increment in the viscosity of DNA.^65^ Reciprocally, a partial and/or non-classical intercalation of ligand could bend (or kink) the DNA helix, reducing its effective length and, simultaneously, its viscosity.^74,75^ The values of (*η*/*η*_0_)^1/3^ were depicted against [complex]/[DNA] that *η* and *η*_0_ are the relative viscosities of DNA in the presence and absence of 1, respectively (Fig. S6†). The plot of Fig. S6† reveals that 1 shows relatively small changes in DNA viscosity, indicating that they bind weakly to DNA, which is consistent with DNA groove binding suggested above.^76^

#### 3.6.3. CD spectral studies

**Fig. 10:**
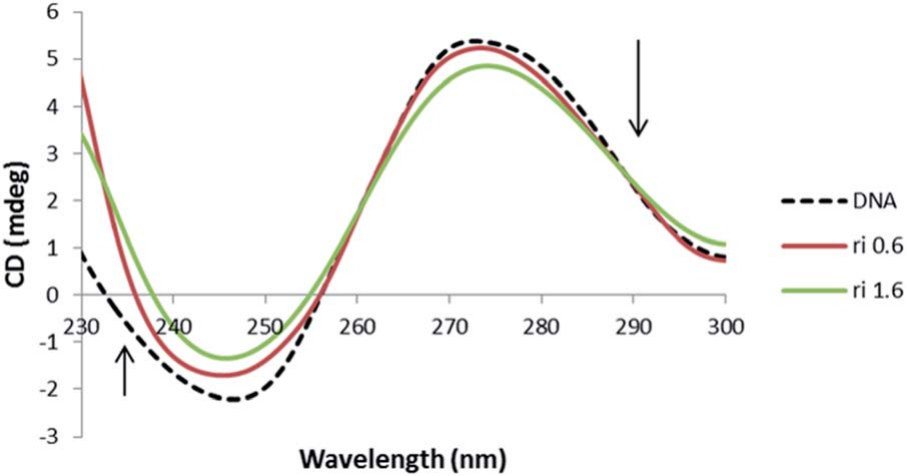
CD spectra of DNA (5 × 10^−5^ M) in 10 mM Tris–HCl buffer, in the presence of increasing amounts of 1 (r_i_ = [complex]/[DNA] = 0.0, 0.6, 1.6).

Due to the sensitivity of CD signal to subtle variation of DNA chiral conformation, CD bands are a useful technique to follow the complex–DNA interaction *via* investigating the changes in the DNA morphology. In particular, B-DNA shows two conservative CD bands in the UV region: a positive band at 278 nm due to base stacking and a negative band at 246 nm because of the right-handed helicity. Both the bands are quite sensitive to the interaction mode with small molecules. Since different DNA structures have different CD spectra, this technique is a powerful procedure to understand the conformational changes of DNA. The CD spectra of DNA in the presence of 1 were illustrated in Fig. 10.

The CD spectra of DNA with different values of the compound exhibit decreases in the positive peak and also decreases in the negative band, which clearly indicates a non-classical intercalation between the complex and DNA.^70^ Decrease in molar ellipticity at these bands (275 nm and 248 nm) suggest the distortion in native conformation of B-DNA due to its interaction with the complex 1. These spectral variations show the presence of some C form DNA features in native conformation of DNA upon interaction. Moreover, reduction in 248 nm CD band is considered a key marker of C form of DNA.^77,78^ However, it seems that the perturbation in DNA conformation is limited to few base pairs because when complete B to C transition occurs, then the CD band at 248 nm shows a 66% decrease in its intensity. However, not much decrease was observed in the concerned band (40.1%). Therefore, there are possibilities for the formation of an intermediate form of DNA having features of both B and C conformation. Similar results have been observed in the case of cationic lipid and neutral lipid binding with DNA, which could be ascribed to a non-cooperative augment in DNA.^79,80^ Moreover, increase in winding angle (or decrease in propeller twist) causes widening in DNA groove that enables proper positioning of small molecules in the groove pocket.^81^

#### 3.6.4. Fluorescence spectra

Fixed amounts (2.5 × 10^−5^ mol L^−1^) of 1 were titrated with increasing amounts of CT-DNA. The complex emits luminescence in Tris–HCl buffer with two maximums that appear at 357 and 408 nm. The fluorescence titrations spectra of 1 in the absence and presence of CT-DNA at 298 K are given in Fig. S7.† The fluorescence intensity of 1 decreased with an increase in CT-DNA concentration. As shown in Fig. S7,† the fluorescence intensity of the compound is quenched steadily with the increasing concentration of the CT-DNA. Similar results were observed for [Cu(adefovir)_2_Cl_2_] complex that interacted with DNA by groove binding.^71^ This phenomenon of the quenching of luminescence of 1 by DNA may be ascribed to the photoelectron transfer from the guanine base of DNA to the excited levels of the complex.^82,83^

##### 3.6.4.1. Fluorescence studies

Quenching is carried out by different mechanisms, which are usually classified as dynamic and static. Dynamic quenching refers to a process in which the fluorophore (here the complex) and the quencher (DNA) come into contact during the transient existence of the exited state, but static quenching refers to fluorophore–quencher complex formation. In general, dynamic and static quenching can be known by their differing dependence on temperature and excited-state lifetime. The dynamic quenching process increases with an increase in the temperature because upon this condition the compounds move faster causing more collision probability. As the formation of composite is responsible for the static quenching, an increase in the temperature diminishes the stability of the composite and causes the fluorescence quenching to descend.^84^ Since in both cases the fluorescence intensity is related to the concentration of the quencher (DNA), the quenched fluorophore can be used as an indicator for the quenching agent.^85^ Fluorescence quenching is described by the Stern–Volmer equation (eqn (2)).

2 *F*_0_/*F* = 1 + *k*_q_*τ*_0_ [Q] = 1 + *k*_sv_ [Q]

where *F*_0_ and *F* represent the fluorescence intensities in the absence and in the presence of quencher, *k*_q_ is the quenching rate constant of the biomolecule, *k*_SV_ is the dynamic quenching constant, *τ*_0_ is the average lifetime of the molecule without quencher and [Q] is the concentration of quencher (here CT-DNA). Of course, these assumptions should be modified by considering the point that quenching can occur through different mechanisms: the inner filter effect, collisional quenching, and binding-related changes in fluorescence. Thus emission needs to be corrected for inner filter effect by the following equation:

3 *F*_cor_ = *F*_obs_ × e^(Aex+Aem)/2^

That *F*_cor_ and *F*_obs_ are the fluorescence intensities corrected and observed, respectively. Moreover, *A*_ex_ and *A*_em_ are the absorption of the system at the excitation and the emission wavelength, respectively. Then the *F*_cor_ is used in eqn (2). The results are shown in Table 6. In this experiment, with an increase in the temperature from 283 K to 310 K, the Stern–Volmer constant (*k*_sv_) decreased from (3.59 ± 0.02) ×10^3^ M^−1^ to (2.70 ± 0.02)×10^3^ M^−1^ for 1 (Fig. S8† and Table 6). It means that the quenching mechanism in the case of 1 is static. Also, *k*_q_ is much larger than 2.0 × 10^10^ L mol^−1^ s^−1^, suggesting a static quenching process. The binding constant (*K*_b_) for the complex formation between 1 and DNA was assayed using eqn (4):^86,87^

4 1/Δ*F* = 1/ (*K*_b_. Δ*F*_0_)[Q] + 1/Δ*F*_0_

Where Δ*F* is fluorescence intensity change, *K*_b_ is the binding constant, and Δ*F*_0_ is maximum fluorescence intensity change. The binding constant can be obtained by plotting 1/Δ*F vs.* 1/[Q] and then dividing intercept by slope.

**Table 6:** Stern–Volmer dynamic quenching constant of 1 to CT-DNA at different temperatures

*T* (K)	*k*_SV_(±0.02)×10^−3^	*k*_q_ (±0.02) × 10^−11^	*R* ^2^
283	3.59	3.59	0.99
298	3.15	3.15	0.99
310	2.70	2.70	0.98

In the present study, the binding constants of 1 were obtained at various temperatures (Table 7). The value of *K*_b_ for 1 at room temperature is comparable to *N*,*N*-bis (3β-acetoxy-5α-cholest-6-yl-idene)hydrazine (4.7 × 10^3^ M^−1^)^88^ and resistomycin (3.23 × 10^3^ M^−1^)^89^ which binds to DNA in an groove binding mode. The plot of log *K*_b_*versus* 1/*T* (Fig. S9†) allows to acquire the enthalpy (Δ*H*), entropy (Δ*S*) (Eq. (5)) and free energy (Δ*G*) change by the van't Hoff equation, (eqn (6)), assuming that the enthalpy change (Δ*H*) is independent of temperature over the range of applied temperatures.

5 ln *K*_b_ = −Δ*H*/*RT* + Δ*S*/*R*

6 Δ*G* = Δ*H* − *T*Δ*S*

**Table 7:** The binding constants and thermodynamic parameters for the copper complex-DNA at different temperatures

*T* (K)	*K*_b_(±0.02) (M^−1^)	Δ*G* (±0.3) (kJ mol^−1^)	Δ*H* (kJ mol^−1^)	Δ*S* (J mol^−1^K^−1^)
283	1.09 × 10^3^	−16.4	—	—
298	3.74 × 10^3^	−20.5	59.6 ± 0.3	268.8 ± 0.3
310	1.00 × 10^4^	−23.7	—	—

Table 7 exhibits the thermodynamic values of 1. According to the data of enthalpy changes (Δ*H*) and entropy changes (Δ*S*), the model of interaction between the complex and biomolecule like DNA can be concluded: (1) Δ*H* > 0 and Δ*S* > 0, hydrophobic forces; (2) Δ*H* < 0 and Δ*S* < 0, van der Waals interaction and hydrogen bonds; (3) Δ*H* < 0 and Δ*S* > 0, electrostatic interactions.^89^ It can be seen that the negative value of Δ*G* revealed the DNA interaction process is spontaneous; the positive Δ*H* and Δ*S* values indicated that hydrophobic forces play main roles in binding the 1 to DNA.

## 4. Conclusions

The work described in this paper involved the synthesis and characterization a binuclear copper(ii) Schiff base complex, [Cu_2_ L_2_^−^ (NO_3_)_2_]·2CH_3_OH (1) from reaction of 2,6-diformyl-4-methoxy phenol, p-methyl aniline and Cu(NO_3_)_2_·2H_2_O in methanol solvent. The complex was characterized by FT-IR, elemental analysis (CHN), UV-vis spectroscopy and molar conductivity measurement. Furthermore, structure of 1 was determined by single crystal X-ray analysis. This complex is neutral and found to have a distorted octahedral geometry with the six donor atoms (N_2_O_4_) of the ligand. The crystal structure and Hirshfeld surface analysis represent the presence of various intermolecular interactions such as H⋯H, H⋯N, H⋯O, H⋯C and C⋯C in assembling the molecules of 1 into a 3D supramolecular network. The 2D fingerprint plots of 1 show that the H⋯H interactions have the largest share of the total intermolecular interactions with contributions of 53.7%. Also, the shape index function accompanied by its 2D fingerprint plot indicate the presence of the π⋯π interactions (5.8%) in the crystal lattice of compound 1. Moreover, the existence of two 1D helical chains in 1 was observed, one along the *b* axis and other one along the perpendicular to *ac* plane (b* axis) *via* C–H⋯O and C–H⋯N hydrogen bonds. This complex exhibited moderate antibacterial activity against the three Gram-positive bacteria: *Staphylococcus aureus* ATCC 25923, *Enterococcus faecalis* ATCC 23212, *S. epidermidis* ATCC 34384, and against the three Gram-negative bacteria: *Escherichia coli* ATCC 25922, *Pseudomonas aeruginosa* ATCC 27853 and *Klebsiella pneumonia* ATCC 70063. Finally, DNA binding of 1 was investigated using UV-vis spectroscopy, fluorescence quenching, CD spectrum and viscosity methods. Fluorescence quenching and UV-Vis spectroscopy results represent that 1 binds to CT-DNA with binding constant of 1.3 ± 0.2 × 10^4^ L mol^−1^. The results of spectroscopic techniques suggested that 1 binds to CT-DNA through groove binding.

## Conflicts of interest

There are no conflicts to declare.

## Supplementary Material

RA-012-D2RA00719C-s001

RA-012-D2RA00719C-s002
