# Supplementary material for: Template synthesis, DNA binding, antimicrobial activity, Hirshfeld surface analysis, and 1D helical supramolecular structure of a novel binuclear copper(ii) Schiff base complex
Source: RSC Adv. 2022 May 5;12(22):13580–92. doi: 10.1039/d2ra00719c (PMC9069724; doi:10.1039/d2ra00719c)
Supplement: RA-012-D2RA00719C-s001 [file RA-012-D2RA00719C-s001.pdf]

Electronic Supplementary Material (ESI) for RSC Advances.
This journal is © The Royal Society of Chemistry 2022

Supplementary Material

Template synthesis, DNA binding, antimicrobial activity, Hirshfeld surface analysis, and 1D helical supramolecular structure of novel binuclear copper(II) Schiff base complex

Hamid Goudarziafshar[1,*], Somaieh Yousefi[2], Yunes Abbasi Tyula[2,*], Michal Dušek[3], Václav Eigner[3]

Table S1. List of abbreviations used in the manuscript with explanations.

Abbreviation	Meaning
2D	Tow dimentional
3D	Three dimentional
CT-DNA	Calf thymus DNA
Tris-HCl	Tris(hydroxymethyl)aminomethane hydrochloride
CD	Circular dichroism
HS	Hirshfeld Surface
FP	fingerprint plots
CIF	Crystallographic Information File
rvdW	van der Waals radii
DMSO	Dimethylsulfoxide
Dimethylformamide	DMF
EtOH	Ethanol
NA	Nutrient agar
MIC	Minimal inhibition concentration
ΔH	Enthalpy changes
ΔS	Entropy changes

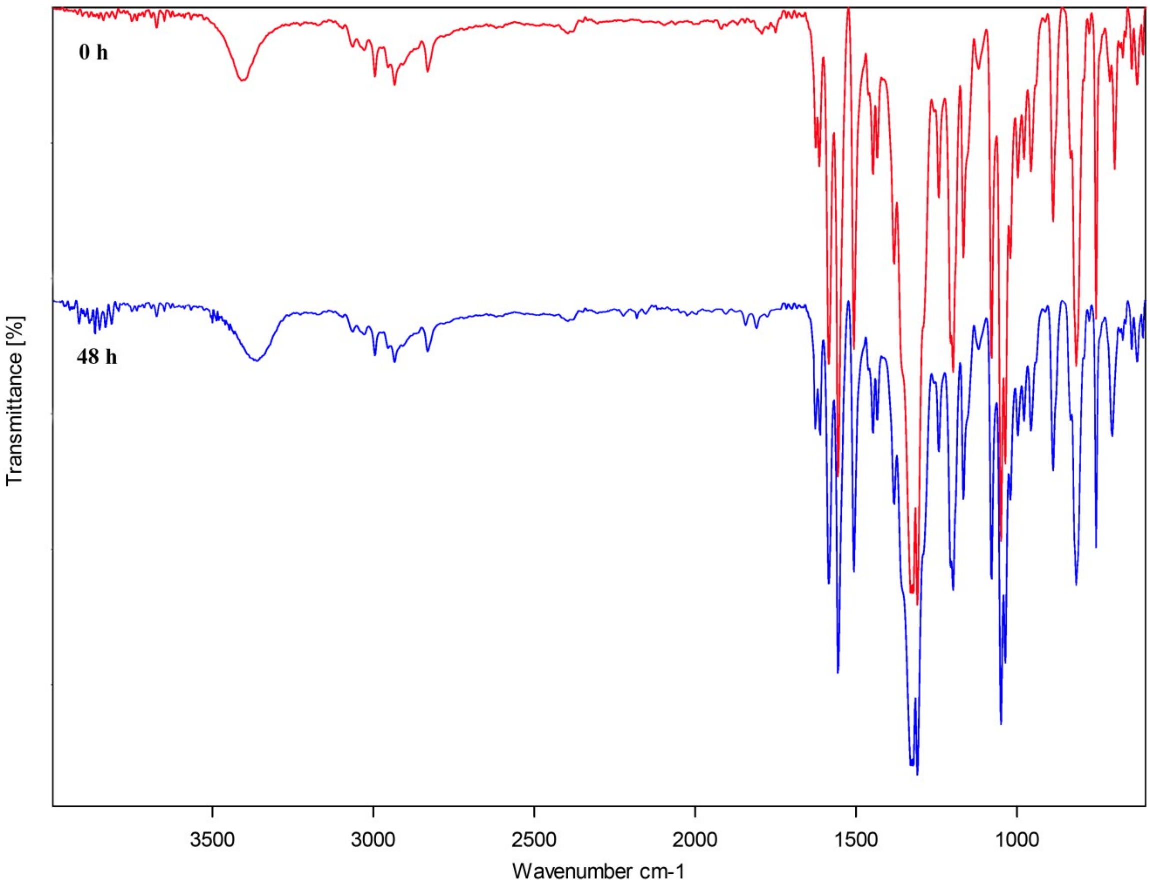

Fig. S1. Solution stability of 1 in DMSO over 48 h examined by FT-IR spectra

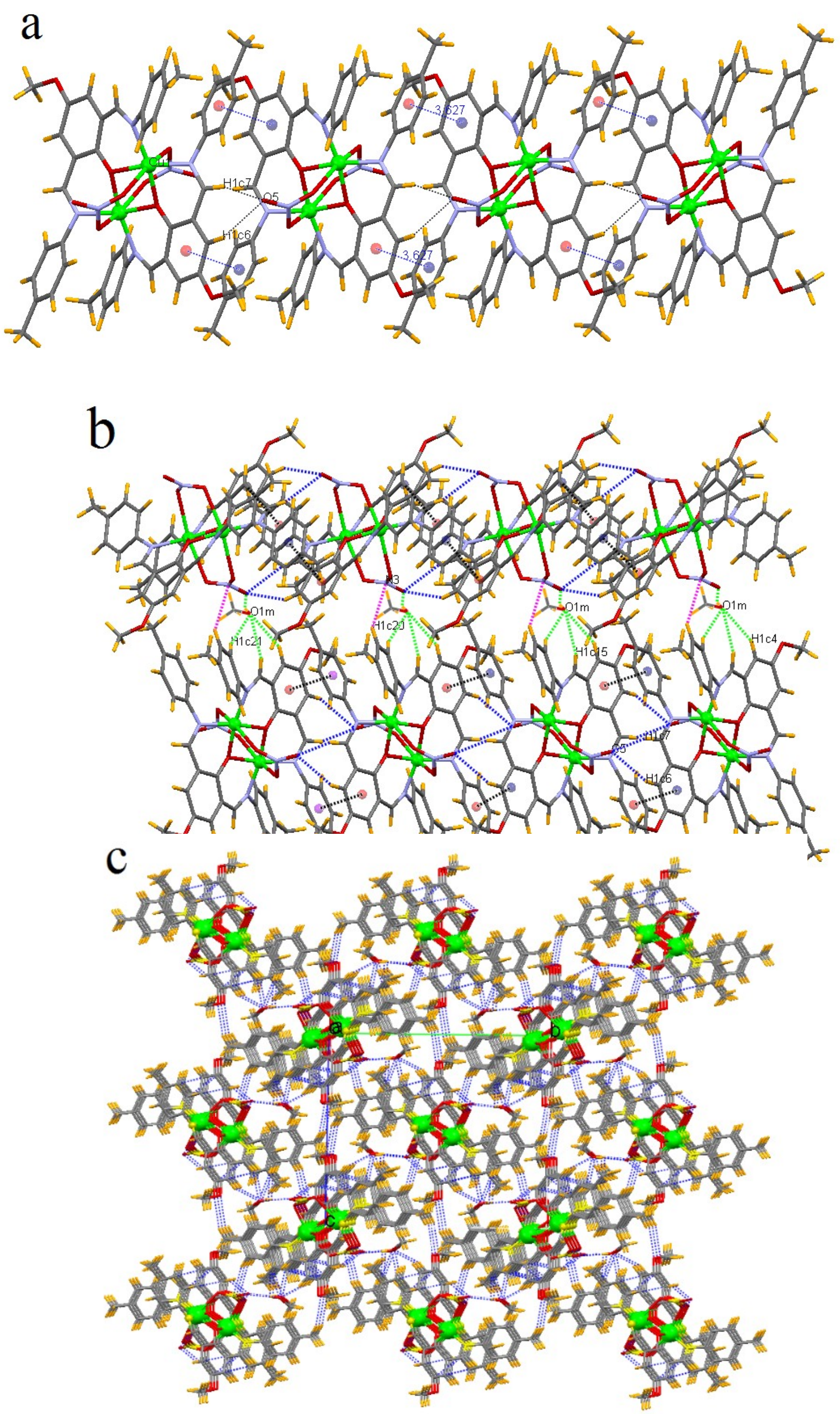
a
Cu1
H1c7
O5
H1c6
3.627
3.627
b
O1m
H1c21
H1c20
O1m
H1c15
O1m
H1c4
H1c7
O5
H1c6
c

Fig. S2. (a) 1D supramolecular structure of **1**, (b) 2D supramolecular structure of **1**, (c) 3D supramolecular structure of **1**, showing the molecules are linked by intermolecular hydrogen bonds and $\pi \ldots \pi$ interactions which are shown as dashed lines.

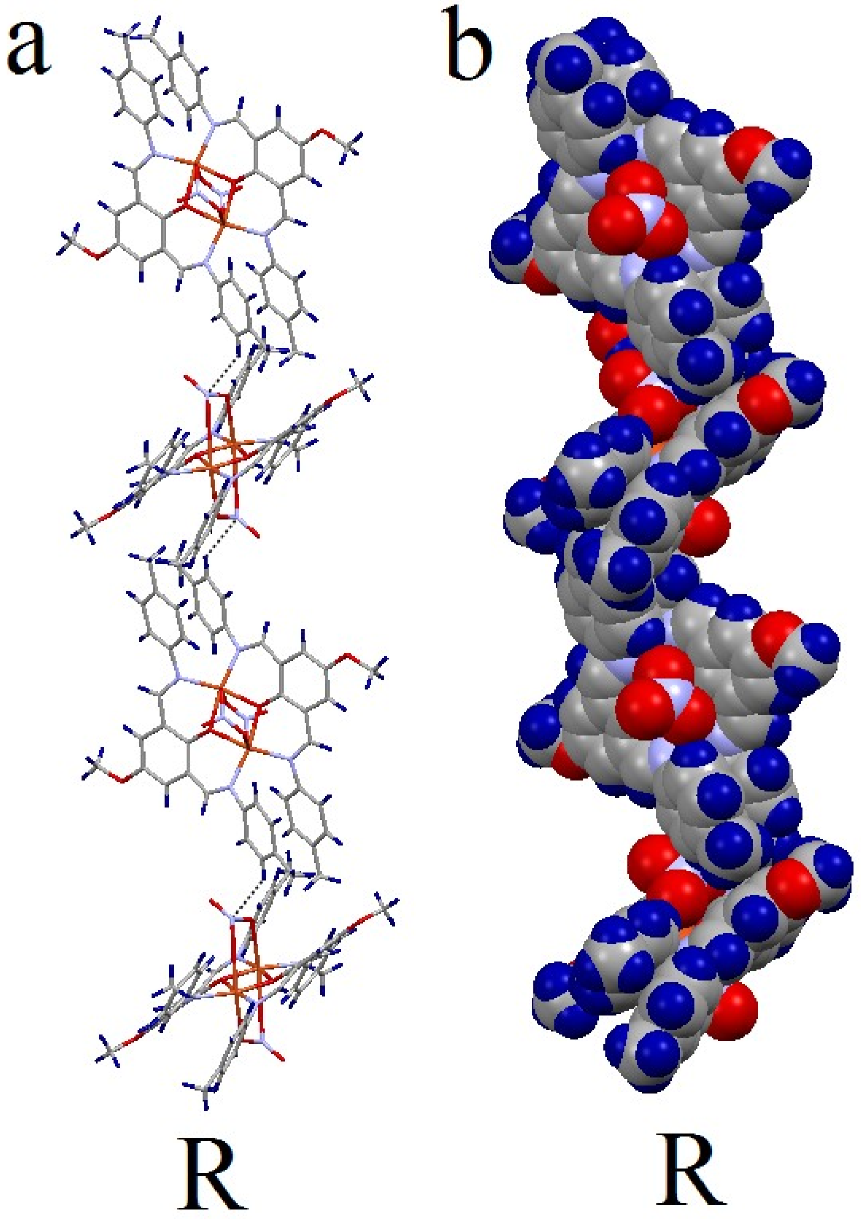

Fig. S3. (a) Hydrogen-bonds network. (b) Space-filling view of the 1D right-handed (R) helical chains along b* axis.

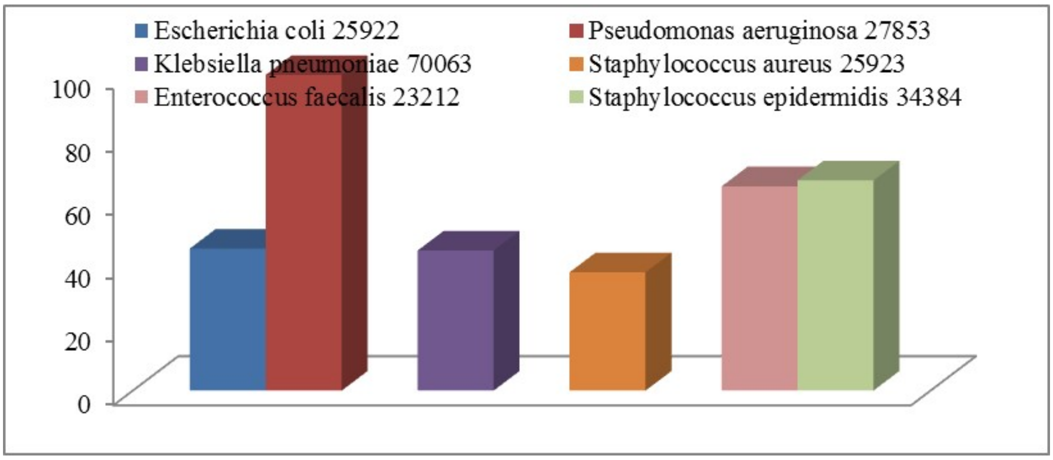

(120-200% inhibition= excellent activity, 90-100% inhibition= good activity, 75-85% inhibition= moderate activity, 50-60% inhibition= significant activity, 20-30% inhibition= negligible activity and no activity).[46]

Fig. S4. Percentage of inhibition of **1**

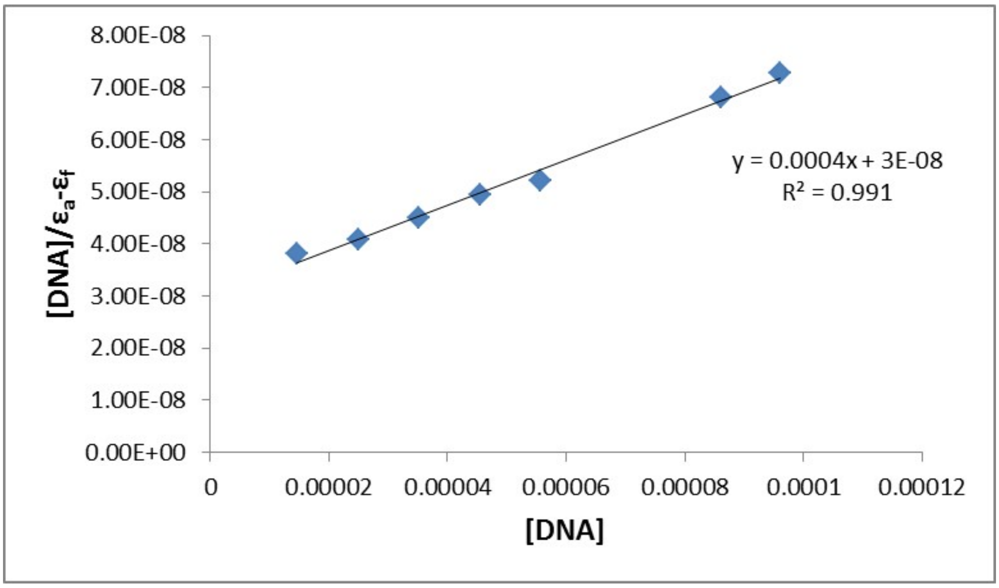

Fig. S5. Plot of [DNA]/(ε_a–ε_f) vs [DNA] for **1**.

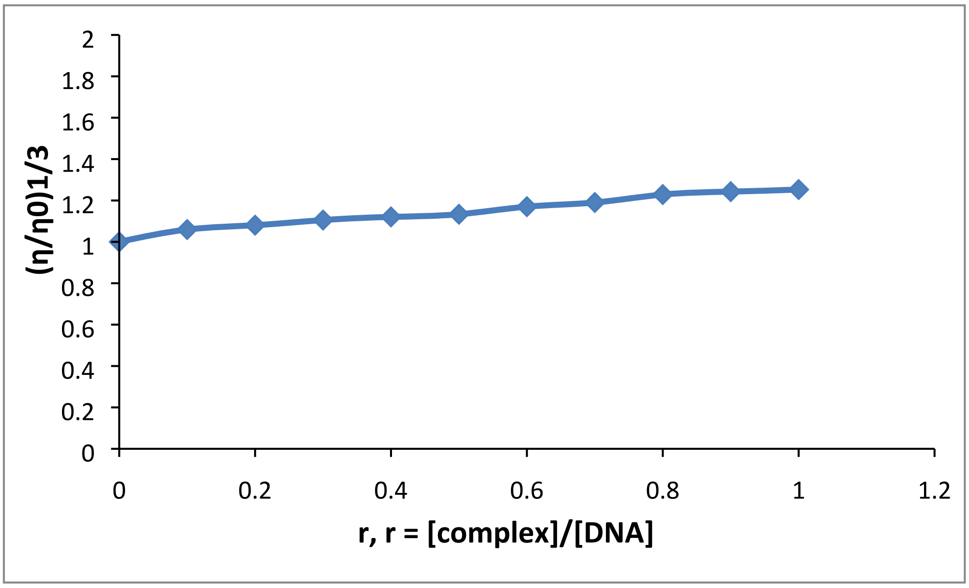

Fig. S6. Effect of increasing concentration of 1 on the relative viscosity of CT-DNA at 25 ºC.

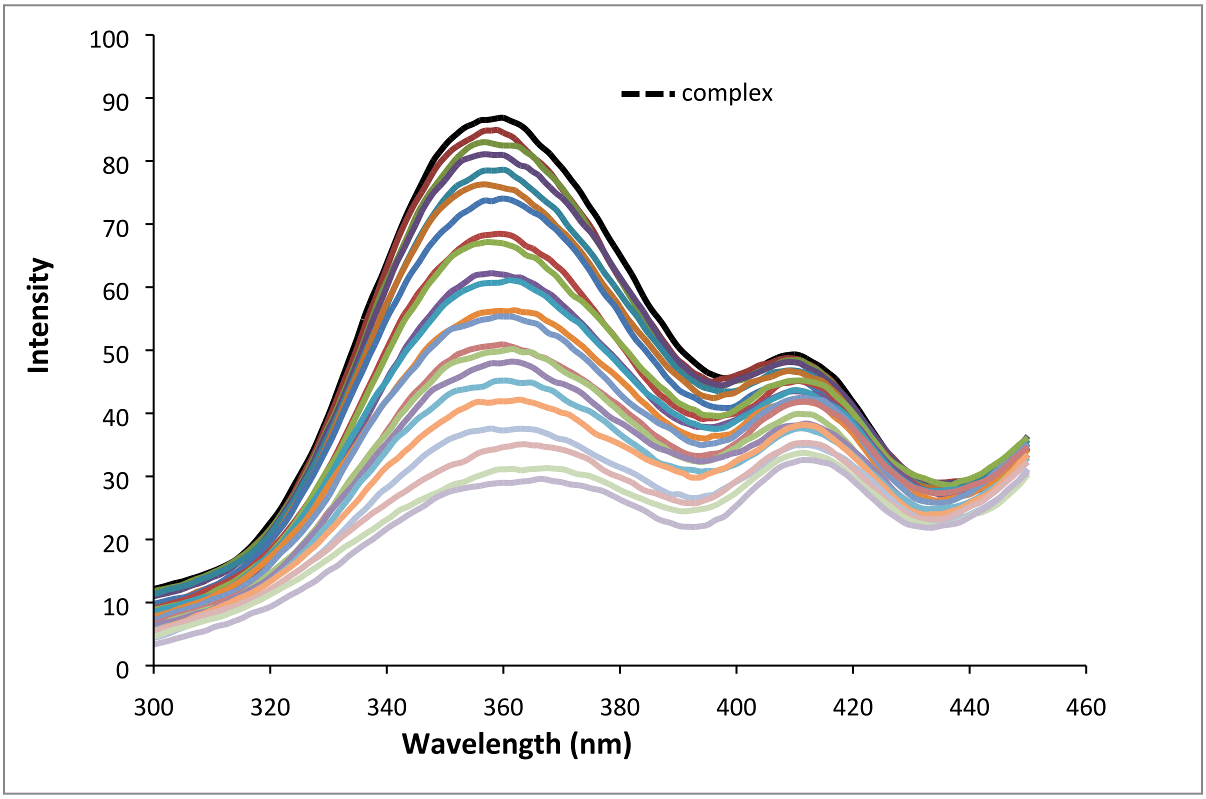

Fig. S7. The fluorescent spectral characteristics of the copper complex–DNA at 298 K. The complex concentration: 2.5×10^{-5} M at pH 7.4 Tris-buffer. DNA concentrations: 0.0 - 5×10^{-4} M.

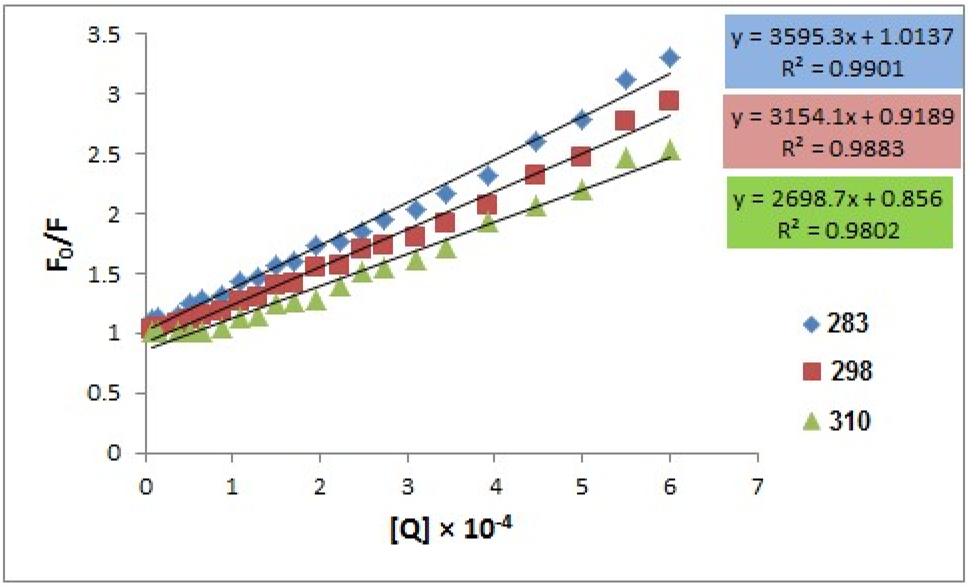

Fig. S8. The plots of F_0/F versus [Q] for calculating K_{sv} for **1**.

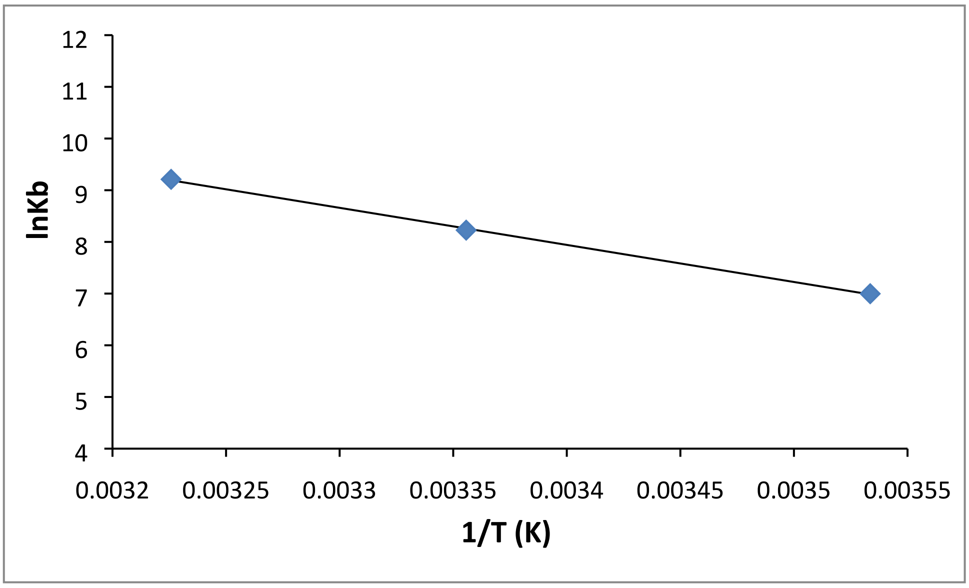

Fig. S9. The plot of lnK_b versus 1/T for **1**.
